# Evaluation of Gilthead Seabream (*Sparus aurata*) Immune Response after LCDV-Sa DNA Vaccination

**DOI:** 10.3390/ani11061613

**Published:** 2021-05-29

**Authors:** Rocío Leiva-Rebollo, Dolores Castro, Patricia Moreno, Juan J. Borrego, Alejandro M. Labella

**Affiliations:** Departamento de Microbiología, Instituto de Biotecnología y Desarrollo Azul (IBYDA), Universidad de Málaga, 29071 Málaga, Spain; rocioleiva@uma.es (R.L.-R.); patriciamgarcia@uma.es (P.M.); jjborrego@uma.es (J.J.B.); amlabella@uma.es (A.M.L.)

**Keywords:** DNA vaccine, lymphocystivirus, immune response, gene expression, gilthead seabream

## Abstract

**Simple Summary:**

Lymphocystis disease is the main viral pathology in gilthead seabream aquaculture. Currently, there are no treatments or vaccines to control this disease, thus our main goal was to construct a DNA vaccine that can be used in the future to stop the spread of this pathology in sea farms. The vaccine consisted of a plasmid DNA that contains a known viral gene. Once it was established that the vaccine drives the expression of the antigenic viral protein in fish, vaccination experiments were conducted to determine if the vaccinated fish become protected against the viral infection. In addition, the immune response triggered by the vaccine was also evaluated in order to understand the mechanisms underlying such protection. The obtained results showed that in vaccinated fish an activation of several genes relating to both the inflammatory process and the mucosal immunity were produced, as well as specific anti-viral antibodies. Although limited, our results deserve further investigation to assess the efficacy of the vaccine in bigger fish populations and to confirm the mode of action of the vaccine.

**Abstract:**

Lymphocystis disease is the main viral pathology reported in gilthead seabream. Its etiological agent is *Lymphocystis disease virus 3* (LCDV-Sa), genus *Lymphocystivirus*, family *Iridoviridae.* There are no effective treatments or vaccines for LCDV control, thus the main aim of this study was to develop a DNA vaccine, and to evaluate both the protection conferred against LCDV-Sa infection and the immune response in vaccinated fish. The vaccine was constructed by cloning the *mcp* gene (ORF LCDVSa062R) into pcDNA3.1/NT-GFP-TOPO. Two independent vaccination trials were conducted. In the first one, 5–7 g fish were intramuscularly injected with the vaccine (pcDNA-MCP) or the empty-plasmid, and the distribution and expression of the vaccine was investigated. Furthermore, vaccinated fish were challenged with LCDV-Sa in order to access the protective capacity of the vaccine. In the second trial, 70–100 g fish were vaccinated as specified, and the immune response was evaluated analyzing the expression of 23 immune-related genes and the production of specific antibodies. The results showed that the vaccine triggers an immune response characterized by the overexpression of genes relating to the inflammatory process, but not the innate antiviral immunity relating to type I IFN (interferon), and also induces the production of specific neutralizing antibodies, which could explain the protection against LCDV-Sa in vaccinated fish.

## 1. Introduction

Lymphocystis disease (LCD) is the main viral pathology associated with the production of gilthead seabream (*Sparus aurata*), one of most important marine fish species in the Mediterranean and South Atlantic aquaculture [1,2]. This pathology is characterized by the appearance of small pearl-like nodules, with papilloma-like appearance, on the skin and fins of affected fish [3,4]. Although described as a self-limited disease, LCD outbreaks may provoke important economic losses in the aquaculture sector [5], with sporadic episodes of high mortality in juvenile fish also being reported [1,6,7]. There are no effective treatments for LCDV control, and LCD prevention in fish farms relies on general prophylactic practices such as reduced stocking density or virologic control of fish and/or live food to be introduced in farm facilities [3,8,9,10].

The etiological agent of LCD in gilthead seabream is *Lymphocystis disease virus 3* (LCDV-Sa), a recently recognized species in the genus *Lymphocystivirus*, family *Iridoviridae* [11,12]. LCDV-Sa subclinical infections are frequently detected in gilthead seabream farms, even in those where lymphocystis outbreaks have not been reported, indicating that this fish species is a common LCDV-carrier [9,13,14,15]. Furthermore, it has been demonstrated that LCDV-Sa establishes an asymptomatic infection in recovered animals, which may extend for at least two months after recovery [15,16]. This highlights the need to establish specific prevention measures to limit this viral infection.

Vaccination is one of the most important measures for the control of fish diseases, mostly those of viral etiology [17,18,19]. DNA vaccines offer advantages over conventional vaccines, including their relatively easy and low-cost production and their stability, and additionally they induce not only cellular and humoral adaptive immunity, but also some innate immune responses [19,20,21,22].

Two DNA vaccines have been developed to prevent LCD in Japanese flounder (*Paralichtys olivaceus*). Both vaccines, based on the major capsid protein (MCP) gene of *Lymphocystis disease virus 2* (LCDV-C), induced an immune response in this fish species, although data on the protection provided by the vaccines are very limited [23,24,25]. However, the variability in the *mcp* gene exhibited by members of the genus *Lymphocystivirus* [12] made vaccines based on LCDV-C unsuitable to confer protection against LCDV-Sa.

The aim of this study was to develop a DNA vaccine containing the gene encoding the MCP of LCDV-Sa, analyze its distribution and expression levels in gilthead seabream, and evaluate the protection conferred against LCDV-Sa infection. In addition, the induction of the immune response of gilthead seabream after vaccination was evaluated by quantifying the expression of 23 immune-related genes in head kidney, a primary immune organ in fish, and intestine, and determining the production of specific antibodies.

## 2. Materials and Methods

### 2.1. Fish

Gilthead seabream specimens were obtained from IFAPA centre El Toruño (El Puerto de Santa María, Spain), belonging to a single cohort. Two vaccination trials were conducted, using fish weighing 5–10 g and 70–100 g. Before each trial, 10 fish were randomly collected and analyzed by real-time PCR (qPCR) [14]. Fish were acclimatized for two weeks before starting each experiment. The water temperature was 22 ± 1 °C and the salinity was 35–37 g/L. The fish were fed with commercial pellets of the proper size (Skretting, Burgos, Spain) once per day (1% fish biomass) and maintained under natural photoperiod conditions. Fish were subjected to a 24-h starvation period prior to the experimental procedures.

All procedures were carried out under the Spanish directive (RD 53/2013) for the protection of animals used in scientific experiments and authorized by the Spanish authorities for the regulation of animal care and experimentation (registration number 10-06-2016-102).

### 2.2. Virus and Cell Culture

The LCDV-Sa isolate used in this study was obtained from diseased gilthead seabream as specified by Leiva-Rebollo et al. [26] and titrated in SAF-1 cells using the 50% cell culture infectious dose (TCID_50_) end-point dilution assay as previously described [27].

The presence of infectious viruses in viral suspensions incubated with fish serum (see below) was demonstrated by the integrated cell culture (ICC)-RT-PCR assay described by Valverde et al. [28]. Briefly, semi-confluent SAF-1 cell monolayers in 24-well plates were inoculated in duplicate with the viral suspensions (200 µL per well). Inoculated cells were harvested at 5 d post-inoculation and total RNA was extracted, treated with DNase and used as a template for one-step RT-PCR using primers targeting the *mcp* gene. Finally, RT-PCR products were specifically detected by dot-blot hybridization. The detection limit of this ICC-RT-PCR assay is 0.1 TCID_50_/mL [28].

### 2.3. Vaccine Construction

The complete ORF encoding the viral MCP was amplified by PCR using DNA from the LCDV-Sa isolate as template and primers MCP-F and MCP-R (Table 1), containing both the start and stop codons. The PCR product was cloned into the eukaryotic expression vector pcDNA3.1/NT-GFP-TOPO, following manufacturer’s instructions (Invitrogen, Life Technologies Co., Carlsbad, CA, USA). This vector allows the expression of viral MCP as a fusion protein with the green fluorescent protein (GFP) under the early cytomegalovirus promoter. The vaccine construct, named pcDNA-MCP, was used to transform *Escherichia coli* One Shot TOP10 cells (Invitrogen). A clone containing pcDNA-MCP was identified by PCR and verified by Sanger sequencing, using the primers and protocols provided with the cloning kit (NT-GFP Fusion TOPO^®^ TA Expression Kit) (Invitrogen). A re-ligated empty pcDNA3.1/NT-GFP-TOPO plasmid (pcDNA) was used as negative control.

*E. coli* cultures containing pcDNA-MCP or pcDNA plasmids were conserved at −80 °C in LB broth supplemented with ampicillin (100 µg/mL) and glycerol (20%, *v*/*v*). For large scale purification, the EndoFree Plasmid Mega Kit (Qiagen, Hilden, Germany) was used, and the concentration of the purified plasmid was determined by spectrophotometry using a NanoDrop 1000 (Thermo Scientific, Life Technologies Co., Carlsbad, CA, USA). Purified plasmids were conserved at −20 °C until used.

### 2.4. Nucleic acid Extraction and cDNA Synthesis

DNA extractions from fish organs (10–20 mg) were carried out using E.Z.N.A. Tissue DNA Kit (Omega Bio-Tek Inc., Norcross, GA, USA) following manufacturer’s recommendations. DNA samples were resuspended in DNase-free buffer, quantified by spectrophotometry (NanoDrop 1000) and stored at −20 °C until used.

To study expression driven by pcDNA-MCP in vaccinated fish, or viral expression in viral challenged vaccinated fish, total RNA from organs (10–20 mg) were extracted using E.Z.N.A. Total RNA Kit (Omega Bio-Tek), and then, cDNA was synthesized from 1 µg of total RNA using Transcriptor First Strand cDNA Synthesis Kit (Roche Diagnostics, Barcelona, Spain). On the other hand, to analyze expression of host immune-related genes, samples (30–50 mg) were processed following the procedure specified by Labella et al. [29]. The cDNA synthesis was carried out using the High-Capacity cDNA Reverse Transcription Kit (Applied Biosystems, Life Technologies Co., Carlsbad, CA, USA) and 2 µg of total RNA. All RNA samples were resuspended in nuclease-free water and treated with RNase-free DNase I (Sigma–Aldrich, Merk, Darmstadt, Germany) following the manufacturer’s instructions and quantified by spectrophotometry as specified above. After DNase treatment, the absence of residual DNA was confirmed by qPCR. cDNA samples were stored at −20 °C until used.

### 2.5. In Vivo Distribution and Expression of pcDNA-MCP

Gilthead seabream specimens (5–10 g weight) were separated into three experimental groups (50 fish each) and maintained in 100 L-capacity opaque tanks with independent recirculation systems. Fish were anesthetized with MS-222 (Sigma–Aldrich), and intramuscularly injected with 100 μL of phosphate-buffered saline (PBS) containing 1 µg/g fish of pcDNA-MCP (vaccinated group) or pcDNA (mock-vaccinated group), or 100 μL of PBS (control group). Six fish per group were randomly selected at 7, 14 and 20 d post-vaccination (dpv), and euthanized by a MS-222 overdose. Samples from muscle at the site of injection, head kidney (HK) and caudal fin were aseptically collected, frozen in liquid nitrogen and stored at −80 °C until used. Nucleic acid extractions were performed as specified above, using samples from 3 fish for DNA, and from the other 3 for RNA.

The detection of the plasmid DNA and *mcp* transcripts in vaccinated fish were carried out by nested-PCR (nPCR). PCR assays were performed using a 50-µL final volume reaction containing 10 µL of Green GoTaq Flexi Buffer 5X (Promega Co., Madison, WI, USA), 5 µL deoxynucleotide triphosphates (dNTPs) (Roche Diagnostics), 5 µL of MgCl_2_ 25 mM, 15 pmol/µL of each primer (Table 1), 0.5 µL of GoTaq DNA Polymerase (Promega Co.) and 100 ng of DNA or cDNA generated from 100 ng of the original RNA template. The nPCR were performed using a 50-µL final volume reaction with the same composition specified, except the amounts of MgCl_2_ 25 mM (3 µL) and GoTaq DNA Polymerase (0.25 µL) and the primer pair (Table 1), using 2 µL of the PCR product as template. In both cases, the amplification conditions were: 95 °C for 5 min, 35 cycles (PCR) or 30 cycles (nPCR) at 95 °C for 1 min, 60 °C for 30 s and 72 °C for 90 s, with a final step at 72 °C during 10 min. Amplified products were visualized by 2% agarose gel electrophoresis.

### 2.6. Viral Challenge to Evaluate Vaccine Protection

Twenty-one days after the vaccination trial described above, gilthead seabream specimens from vaccinated and mock-vaccinated groups were anesthetized with MS-222, divided in two subgroups and injected intraperitoneally with 100 µL of the LCDV-Sa stock (10^5^ TCID_50_ per fish) or 100 µL of Leibovitz L-15 medium (Gibco, Life Technologies Co., Carlsbad, CA, USA). Fish in each experimental condition were maintained in 46-L aquaria without recirculation, with a partial water change (1/2 water volume) every 2 or 3 days. Six fish per subgroup were randomly selected at 2 and 10 d post-inoculation (dpi), euthanized by a MS-222 overdose and caudal fin samples (60–100 mg) were aseptically collected and frozen in liquid nitrogen. Samples were ground in liquid nitrogen using a mixer mill MM400 (Retsch, Haan, Germany), and subsequently used for both DNA and RNA extraction.

Viral DNA detection and quantification were carried out by qPCR, using the methodology described in Appendix A. Viral loads were expressed as copies of viral DNA per milligram of tissue. Viral transcripts were detected by RT-qPCR, following the protocol mentioned above, but using 20-μL final volume reactions and cDNA generated from 100 ng of the original RNA template.

### 2.7. Gilthead Seabream Immune Response after pcDNA-MCP Vaccination

To evaluate the immune response of fish after vaccination, gilthead seabream specimens (70–100 g weight) were separated into three experimental groups, anesthetized with MS-222, and intramuscularly injected, approximately 1 cm below the dorsal fin, with: (1) 10 µg/100 μL of pcDNA-MCP (vaccinated group); (2) 10 µg/100 μL of pcDNA (mock-vaccinated group), and (3) 100 μL of PBS (control group). Animals from each experimental group (40 fish) were distributed into 2 opaque tanks (300 L volume), and tanks were connected to three independent recirculation systems. Five fish per group were randomly selected at 1, 3 and 8 dpv. Samples from the HK and intestine were aseptically collected, frozen in liquid nitrogen and stored at −80 °C until used to evaluate the expression of immune-related genes in the vaccinated fish.

In addition, the production of specific antibodies in the vaccinated fish was also investigated. Five fish from the vaccinated group were sampled 1, 2 and 3 months after vaccination. Blood was extracted from the caudal vein of the anesthetized fish using a 25-gauge needle. After clotting overnight at 4 °C, serum was collected by centrifugation (10,000× *g* 10 min at 4 °C) and stored at −80 °C until used. Sera obtained from 5 fish from the control group sampled 1 month after PBS injection were used as negative control.

#### 2.7.1. Host Gene Expression Analysis in Response to Vaccination

The expression of 23 host immune-related genes and the *mcp* gene carried in the vaccine were analyzed by RT-qPCR using the primer sets and protocols described by Leiva-Rebollo et al. [26]. In short, relative mRNA expression was determined using the 2^−ΔΔCt^ method, with two reference genes (*ef1α* and *actβ*) used to normalize gene expression and samples of the control group (injected with PBS) as calibrator. In the case of the *mcp* gene, relative expression levels in each organ were calculated with the two reference genes already mentioned and 1 dpv samples as calibrator. Data were expressed as fold change (mean ± SEM).

The identification of differentially expressed genes (DEGs) in the two organs analyzed at different times, both in vaccinated and mock-vaccinated fish, was performed by volcano plot construction, using a *p*-value < 0.05 and |log_2_ fold change| > 0.5 as threshold for statistical significance [26].

Expression level of DEGs were compared between vaccinated and mock-vaccinated groups, to determine the genes that were differentially expressed in response to the vaccine, and not the empty plasmid. Finally, a comparative analysis of the transcriptional level of DEGs over time in both organs analyzed was performed.

#### 2.7.2. Specific Antibodies Titration and Neutralizing Antibodies Detection

An enzyme-linked immunosorbent assay (ELISA) was used to detect MCP-specific IgM in the serum of vaccinated fish. For IgM titration, an end-point protocol was carried out using serial two-fold dilutions (from 1:32 to 1:65,536) of serum in PBS.

To obtain the antigen for specific IgM capture, the complete ORF encoding the viral MCP was sub-cloned into the prokaryotic expression vector pGEX-6P-3 (GE Healthcare, Merk, Darmstadt, Germany), and used to transform *E. coli* BL21 cells (GE Healthcare). This vector allows the expression of viral MCP as a fusion protein with the glutathione S-transferase (GST). The expression of the GST-tagged MCP in the bacterial culture was induced by incubation with 0.8 mM IPTG (Sigma-Aldrich) at 37 °C during 2 h. After the induction period, bacterial cells were harvested by centrifugation, lysed by sonication followed by Triton X-100 (Sigma-Aldrich) treatment, and the recombinant protein purified from the supernatant by affinity chromatography using Glutathione Sepharose 4B (GE Healthcare). Standard protocols provided by the manufacturer were used.

For the ELISA analysis, 96-well plates (Nunc Maxisorp) (Thermo Scientific) were coated overnight at 4 °C with 100 μL of the recombinant GST-MCP diluted in 100 mM bicarbonate buffer at a final concentration of 10 μg/mL. After three washes (5 min each) with PBS supplemented with 0.25% Tween 20 (PBS-T), the plates were blocked for 1 h with 300 μL of 0.25% (*w*/*v*) bovine serum albumin in PBS-T, and washed three times with PBS-T. Then, 50 μL of the diluted serum samples were incubated for 2 h, followed by three washes with PBS-T. The plates were incubated with 50 μL rabbit anti-gilthead seabream IgM polyclonal antibody and monoclonal anti-rabbit IgG HRP-conjugated antibody as described above. After a final washing step, 50 μL of TMB liquid substrate was added, followed 10 min later by 50 μL of stop solution. All washes and incubations were made at room temperature, and reagents were obtained from Sigma–Aldrich. Finally, the absorbance was read at 450 nm. Optical density (OD) values of blank controls without fish serum were subtracted for each sample value.

The ELISA positive threshold value was calculated as the mean and standard deviation of the OD observed for negative control sera diluted 1:100. The titer of antibodies specific to MCP was defined as the maximal dilution of the serum with an OD higher than the positive threshold plus the standard deviation multiplied by 3 [30].

A qualitative seroneutralization assay was performed to detect neutralizing antibodies in the serum of vaccinated fish. Three LCDV-Sa suspensions with infectious titers of 10^4^, 10^3^ and 10^2^ TCID_50_/mL were used. Pooled sera from 5 vaccinated fish collected 1, 2 and 3 months after vaccination were diluted 1:32 and 1:64 in Leibovitz L-15 medium. Sera dilutions or L-15 medium (negative control) were incubated with equal volumes of the viral suspensions for 1 h at 20 °C, and the presence of infectious virus was investigated using the ICC-RT-PCR assay previously described.

### 2.8. Statistical Analysis

The statistical tests were carried out using XLSTAT software (Addinsoft Inc., New York, NY, USA). The qPCR data were log-transformed to get normality and homogeneity of variance, performing a Shapiro–Wilk test. Significant differences in viral load or gene expression levels between groups, organs and/or time points were established by using a two-way ANOVA followed by a Bonferroni post-hoc test. A one-way ANOVA followed by Fisher’s LSD test was used to compare a specific organ through time or different organs at a certain point in time. Differences were considered significant when *p* < 0.05.

## 3. Results

### 3.1. Distribution and Expression of the pcDNA-MCP Vaccine

**Figure 1:**
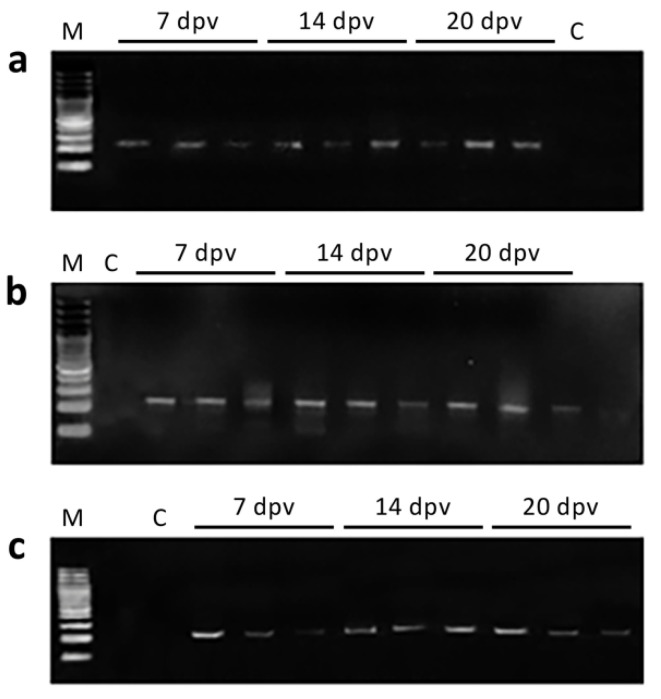
Detection of pcDNA-MCP in vaccinated gilthead seabream at 7, 14, and 20 days post-vaccination (dpv) in different tissues: (**a**) muscle at the site of injection; (**b**) caudal fin; (**c**) head kidney; M, molecular weight marker (100 bp DNA ladder); C, negative control (fish injected with the empty plasmid).

Vaccine plasmid DNA was detected by n-PCR in the muscle, caudal fin and HK of gilthead seabream at 7, 14, and 20 dpv (Figure 1). As expected, no plasmid DNA was detected in samples from the mock-vaccinated and control groups.

**Figure 2:**
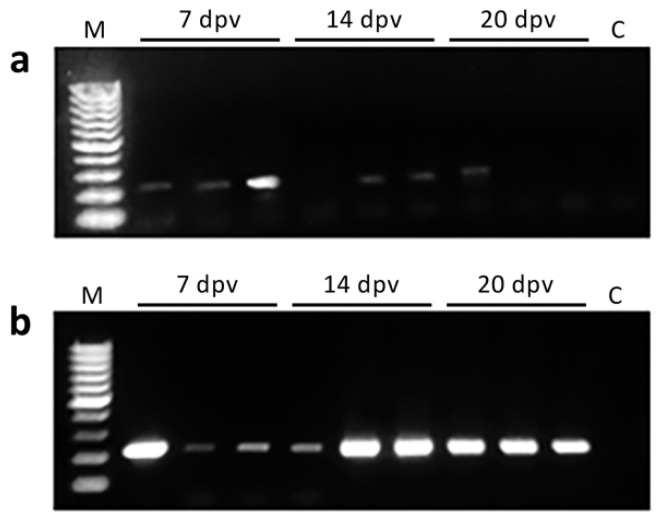
Detection of *mcp* transcripts in vaccinated gilthead seabream at 7, 14, and 20 days post-vaccination (dpv) in (**a**) muscle at the site of injection, and (**b**) head kidney; M, molecular weight marker (100 bp DNA ladder); C, negative control (fish injected with the empty plasmid).

The transcription of the *mcp* gene codified in the vaccine was detected in the muscle of all fish at 7 dpv, in two out of three fish analyzed at 14 dpv, and in one fish at 20 dpv, whereas *mcp* transcripts were detected in the HK in all fish analyzed (Figure 2). No vaccine driven expression was observed in caudal fin samples.

### 3.2. Evaluation of Vaccine Protection

The protection provided by the pcDNA-MCP vaccine was evaluated by viral load quantification in caudal fin, the main LCDV-Sa target organ, after intraperitoneal injection of LCDV-Sa in vaccinated gilthead seabream at 21 dpv. Samples for viral genome detection and quantification were analyzed by the qPCR assay developed in this study. The results regarding the evaluation of the qPCR assay can be found in Appendix B.

**Figure 3:**
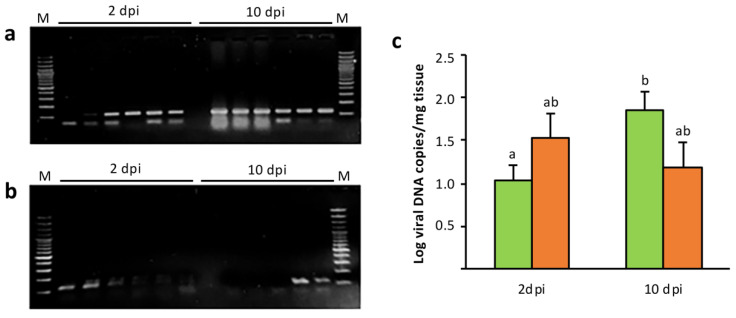
Viral detection in caudal fin samples of gilthead seabream at 2 and 10 days post-inoculation (dpi). LCDV-Sa challenges were carried out in fish 21 days after injection of (**a**) pcDNA (mock-vaccinated fish), and (**b**) pcDNA-MCP (vaccinated fish). M, molecular weight marker (100 bp DNA ladder). (**c**) Viral loads (mean ± SEM) in LCDV-Sa positive samples from mock-vaccinated (green) and vaccinated (orange) fish. Different letters denote significant differences between groups and time points analyzed (*p* < 0.05).

No mortality or signs of disease were registered during the experimental period (10 dpi). In fish injected with the empty plasmid (pcDNA), viral genome was detected in 83.3% of samples analyzed at 2 dpi and in all samples analyzed at 10 dpi (Figure 3a). Estimated viral load was (1.6 ± 0.9) × 10^1^ copies of viral DNA/mg of tissue at 2 dpi. A statistically significant (*p* < 0.05) increase was observed at 10 dpi, with a mean viral load of (1.4 ± 0.7) × 10^2^ copies of viral DNA/mg of tissue (Figure 3c). Only 33.3% of the pcDNA-MCP vaccinated fish analyzed tested positive for LCDV-Sa genome, both at 2 and 10 dpi (Figure 3b), with a mean viral load of (3.0 ± 1.2) × 10^1^ copies of viral DNA/mg of tissue, no statistically significant (*p* < 0.05) differences being observed in viral loads at different days post-infection (Figure 3c). Viral DNA was not detected in any of the caudal fin samples from control fish (injected with L15 medium).

**Figure 4:**
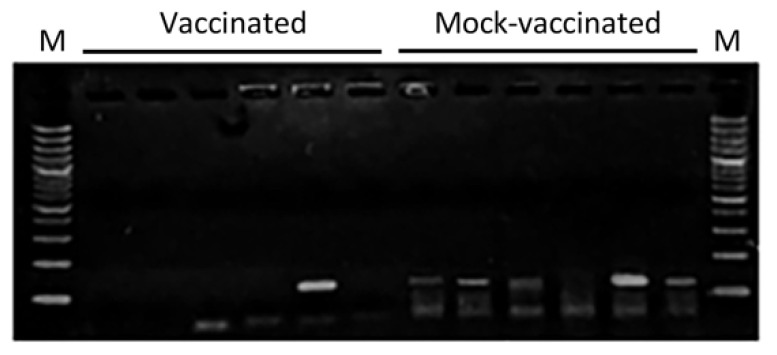
Detection of viral transcripts in caudal fin samples of gilthead seabream 10 days after LCDV-Sa challenge in vaccinated fish (injected with pcDNA-MCP) and mock-vaccinated fish (injected with pcDNA) at 21 days post-vaccination. M, molecular weight marker (100 bp DNA ladder).

Viral gene expression was analyzed as an indicator of productive infection in caudal fin samples collected at 10 dpi. Viral transcripts were detected in five out of six fish injected with the empty plasmid and in one fish injected with the pcDNA-MCP vaccine (Figure 4).

### 3.3. Expression of Immune-Related Genes in Vaccinated Fish

**Figure 5:**
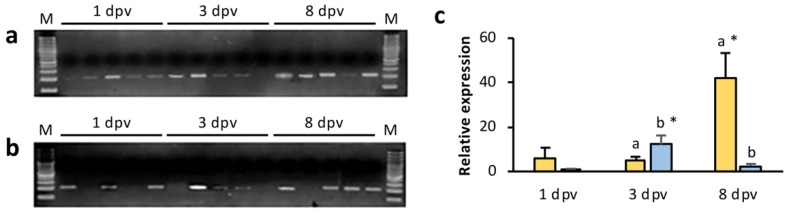
Detection of *mcp* transcripts in vaccinated gilthead seabream at 1, 3, and 8 days post-vaccination (dpv) in (**a**) head kidney, and (**b**) intestine; M, molecular weight marker (100 bp DNA ladder). (**c**) Relative *mcp* expression values (2^−ΔΔCt^) in positive samples of head kidney (yellow bars) and intestine (blue bars) at different times post-vaccination. The mean value of samples collected at 1 dpv in head kidney or intestine was used as calibrator for *mcp* expression in the corresponding organ. Data are expressed as mean ± SEM. Different letters denote significant differences between organs at a time point. Significant differences in an organ analyzed at different times post-vaccination are indicated by an asterisk (*p* < 0.05).

The expression of the *mcp* gene carried in the vaccine was analyzed by RT-qPCR in vaccinated fish. In the HK samples *mcp* transcripts were detected in 80% of the fish analyzed at 1 and 3 dpv, and in all fish analyzed at 8 dpv (Figure 5a). Transcripts were detected in 60% of intestine samples analyzed at 1 and 3 dpv, and in 80% of samples collected at 8 dpv (Figure 5b). The relative *mcp* expression was low in all intestine samples analyzed, with a statistically significant (*p* < 0.05) decrease between 3 and 8 dpv (Figure 5c). The opposite was observed in the HK, with a statistically significant (*p* < 0.05) increase at 8 dpv, reaching a transcription level of 41.8-fold change compared to 1-dpv HK sample used as calibrator (Figure 5c).

To evaluate the immune response in gilthead seabream triggered by the pcDNA-MCP vaccine, a set of 23 genes were analyzed at 1, 3 and 8 dpv in both the HK and intestine. Only two genes were differentially expressed exclusively in head-kidney samples (*mx1* and *mx2*), and seven in the intestine (*irf1*, *isg15*, *casp1*, *nccrp-1*, *tcrβ*, *ighm* and *mhcIIα*).

In the HK samples, 16 genes were differentially expressed, 43.75% of them were down-regulated (*tlr5*, *irf3*, *pkr*, *mx1*, *mx2*, *tnfα* and *il10*) at some point during the experimental period. The *ifn*, *irf9*, *ck3* and *ck10* genes showed an early up-regulation, with *irf9* up-regulated also at 3 dpv but down-regulated at 8 dpv. The *il1β*, *il6* and *c3* genes were up-regulated only a 3 dpv, whereas *tlr9* and *mx3* showed up-regulation at 8 dpv (Table S1).

The number of DEGs in the intestine in response to the vaccine was 20; 80% of them up-regulated at some point during the experiment. The number of up-regulated genes increased over time, with only three genes up-regulated at 1 dpv (*mx3*, *il1β* and *il6*) and 12 genes at 8 dpv. The expression of *irf1*, *irf3* and *isg15* showed a down-regulation at 1 dpv (*irf1* was also down-regulated at 3 dpv); *tnfα* expression was also down-regulated at 1 dpv, although appeared up-regulated at the end of the experimental period. At 3 dpv the up-regulation of *il1β* and *il6* was maintained and extended to *tlr9*, *irf9*, *casp1*, *ck10*, *c3*, *nccrp-1* and *mhcIIα*. Finally, the over expression of *casp1*, *il6*, *ck10*, *c3*, and *mhcIIα* continued until the end of the experimental period, with the other six genes (*tlr5*, *ifn*, *pkr*, *tnfα*, *il10*, *tcrβ* and *ighm*) that appeared up-regulated only at 8 dpv (Table S2).

Differential regulation of some genes was also detected in mock-vaccinated fish (injected with the empty plasmid). Regarding the HK samples, there were 14 DEGs, but only two of them, *irf3* and *tlr9*, were up-regulated at 3 and 8 dpv, respectively, whereas *tlr5* was up-regulated at 3 dpv but down-regulated at the end of the experimental period (Table S1). In the intestine, 4 of the 11 DEGs detected were up-regulated, *mx3* at 1 dpv, *c3* at 3 dpv, and *ifn* and *mhcIIα* at 8 dpv. It should be noted that up-regulation of *mx3* expression in these fish (24.6-fold) was higher than in the vaccinated group (5.3-fold) (Table S2).

**Figure 6:**
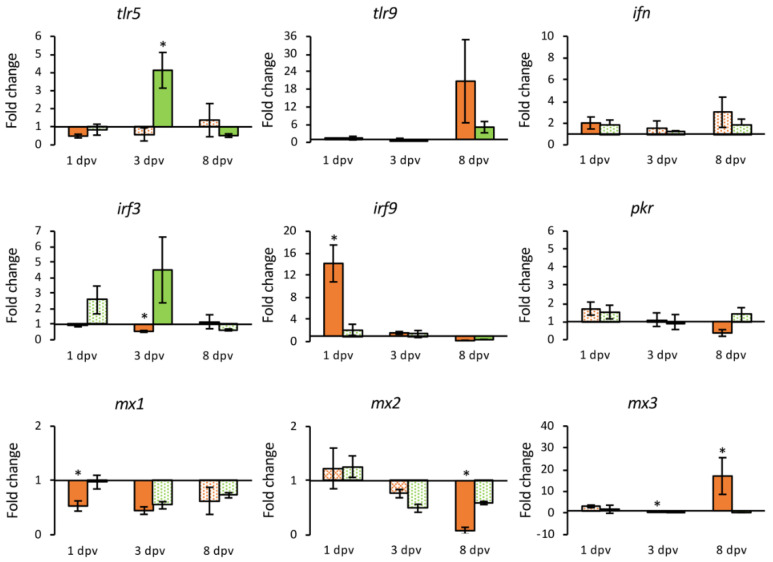
Relative expression levels (2^−ΔΔCt^) of TLR-coding genes and type I interferon related genes in the head kidney from vaccinated (orange) and mock-vaccinated (green) fish at different times post-vaccination (dpv). Differentially expressed genes (DGEs) are represented in solid color. Data are expressed as mean ± SEM (n = 5). Significant differences are indicated by an asterisk (*p* < 0.05).

**Figure 7:**
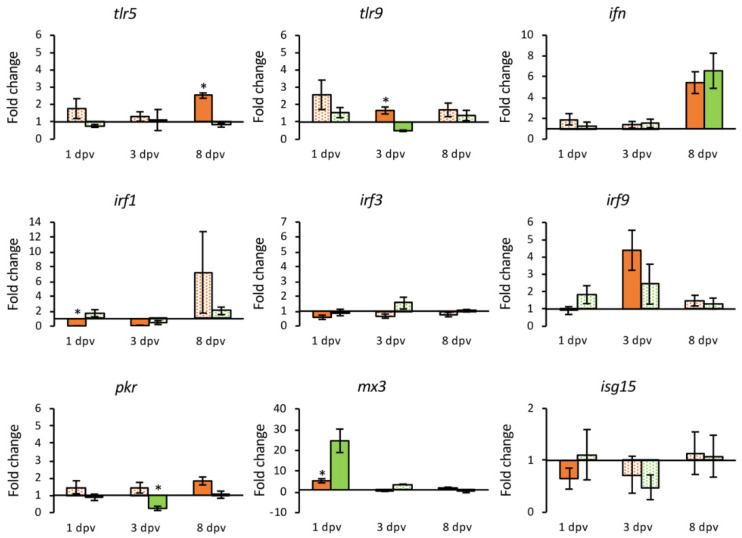
Relative expression levels (2^−ΔΔCt^) of TLR-coding genes and type I interferon related genes in the intestine from vaccinated (orange) and mock-vaccinated (green) fish at different times post-vaccination (dpv). Differentially expressed genes (DGEs) are represented in solid color. Data are expressed as mean ± SEM (n = 5). Significant differences are indicated by an asterisk (*p* < 0.05).

A comparative analysis of the transcriptional level over time between vaccinated and mock-vaccinated groups was carried out to establish the gene expression response that was specific to the vaccine. In those analyses, only DEGs were compared. Gene coding toll-like receptors (TLR) 5 and 9 were differentially expressed in vaccinated fish in both organs, but only in the intestine were they differentially overexpressed compared to fish injected with the empty plasmid. The *inf* gene was differentially up-regulated in both organs in the vaccinated fish but the transcriptional level was similar to the mock-vaccinated fish. The IRF-encoding genes *irf1* and *irf 3* were down-regulated in the intestine and HK, respectively, in vaccinated fish, whereas *irf 9* was up-regulated (14.17-fold change) in the HK at 1 dpv. In the case of interferon induced genes, a regulation of *pkr* was observed in both organs analyzed, although it was not significantly different to that observed in the mock-vaccinated group. The transcription of *mx1* and *mx2* was down-regulated in the HK, and the same was observed for *isg15* in the intestine. The *mx3* gene was up-regulated in both organs, with a seven-day delay in the HK. However, the highest expression level (24.59-fold) was recorded in the intestine from fish injected with the empty plasmid (Figure 6 and Figure 7).

**Figure 8:**
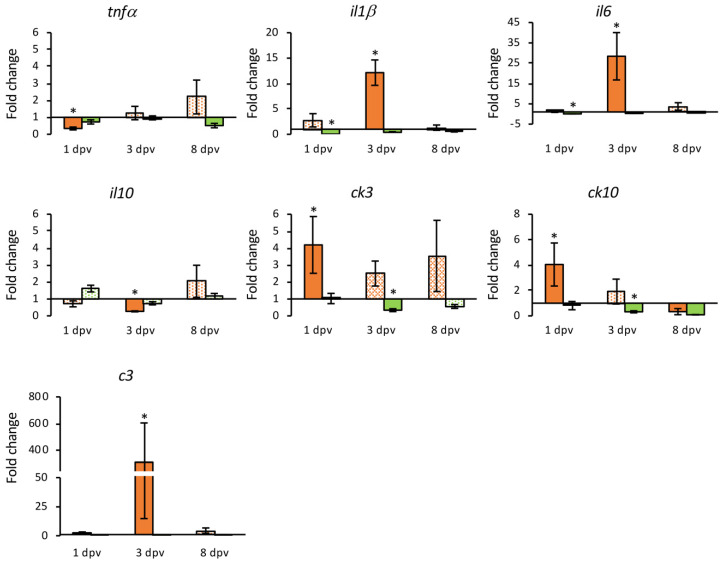
Relative expression levels (2^−ΔΔCt^) of genes coding cytokines and the complement component C3 in the head kidney from vaccinated (orange) and mock-vaccinated (green) fish at different times post-vaccination (dpv). Differentially expressed genes (DGEs) are represented in solid color. Data are expressed as mean ± SEM (n = 5). Significant differences are indicated by an asterisk (*p* < 0.05).

**Figure 9:**
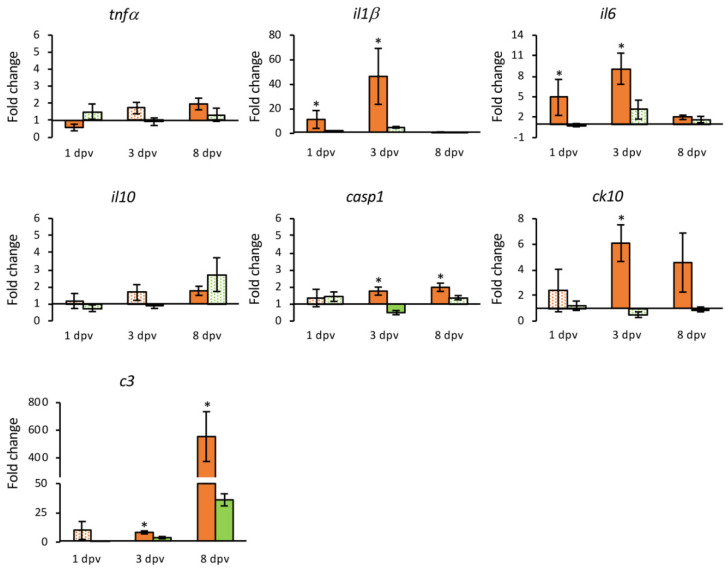
Relative expression levels (2^−ΔΔCt^) of genes coding cytokines and the complement component C3 in the intestine from vaccinated (orange) and mock-vaccinated (green) fish at different times post-vaccination (dpv). Differentially expressed genes (DGEs) are represented in solid color. Data are expressed as mean ± SEM (n = 5). Significant differences are indicated by an asterisk (*p* < 0.05).

Vaccination also induced an important up-regulation of genes associated with pro-inflammatory activity (Figure 8 and Figure 9). The *Il1β* and *il6* genes were up-regulated at 3 dpv in the HK (12.14- and 28.39-fold, respectively), and intestine (46.41- and 9.10-fold), although in the intestine the up-regulation was also observed at 1 dpv (11.34- and 4.90-fold, respectively). The *casp1* gene was up-regulated from the third dpv only in the intestine (fold change 1.76 to 1.98), while an early down-regulation of *tnfα* and *il10* was observed in the HK samples. The chemokines coding genes analyzed (*ck3* and *ck10*) were up-regulated in the HK at 1 dpv (4.19- and 4.04-fold), but only *ck10* presented up-regulation in the intestine at 3 dpv (6.09-fold). The highest expression levels were observed for the complement fraction *c3* gene that was overexpressed in both organs (309.41-fold in the HK at 3 dpv, and 553.65-fold in the intestine at 8 dpv).

**Figure 10:**
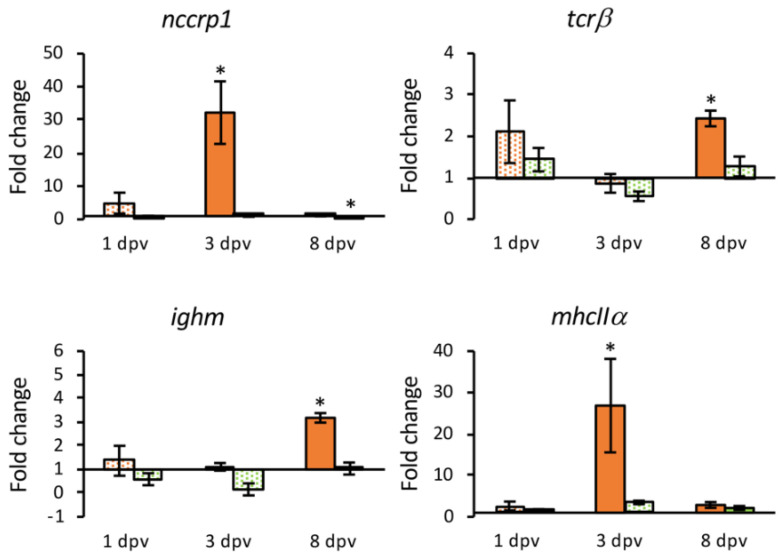
Relative expression levels (2^−ΔΔCt^) of genes coding cell receptor markers in the intestine from vaccinated (orange) and mock-vaccinated (green) fish at different times post-vaccination (dpv). Differentially expressed genes (DGEs) are represented in solid color. Data are expressed as mean ± SEM (n = 5). Significant differences are indicated by an asterisk (*p* < 0.05).

Finally, an overexpression of the cell receptor markers analyzed was observed only in the intestine of vaccinated fish at 3 or 8 dpv. At 3 dpv, *nccrp1* and *mhcIIα* genes were significantly up-regulated (32.22- and 26.85-fold, respectively), whereas the up-regulation of *tcrβ* and *ighm* genes was observed at 8 dpv (2.43- and 3.18-fold) (Figure 10).

### 3.4. Antibody Production in Vaccinated Gilthead Seabream

**Figure 11:**
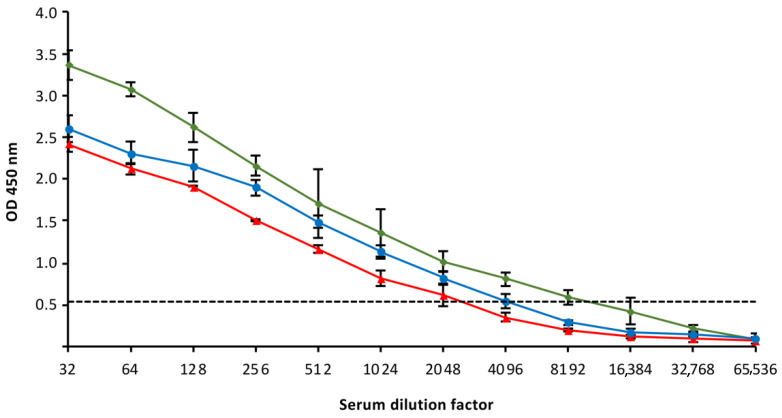
ELISA detection of specific anti-MCP antibodies in gilthead seabream sera collected one (green), two (blue) and three (red) months post-vaccination. Data are expressed as mean ± SD (n = 5). The positive threshold is represented by a dotted line.

The generation of anti-MCP antibodies in the serum from vaccinated fish at 1, 2, and 3 months post-vaccination was evaluated by using an ELISA protocol (Figure 11). Specific antibodies were detected at the three time-points analyzed, with the highest antibody titer (4096) determined at 30 dpv. At 2 and 3 months pv, the estimated antibody titer was 1024.

The neutralizing capacity of the serum from vaccinated fish was also evaluated, using pooled sera from five vaccinated fish at each time-point analyzed and an ICC-RT-PCR assay to determine viral infectivity. No infectious virus was detected after the neutralization assay with a viral suspension of 10^4^ TCID_50_/mL and 1:32 dilution of the serum, which implies a reduction in the infectious titer of at least five orders of magnitude. The dilution 1:64 of the serum produced a complete neutralization of the viral infectivity using the 10^3^ TCID_50_/mL viral suspension.

## 4. Discussion

Currently, there are no effective treatments or commercial vaccines available to prevent LCD outbreaks in fish farms; thus, control measures rely on the selection of LCDV-free broodstock, egg decontamination procedures to prevent virus spread from asymptomatic broodstock to larvae, and the supply of virus-free live food [9,31,32].

DNA vaccines are an effective method for the prevention of infectious diseases, and there are a growing number of experimental vaccines for important viral pathogens in aquaculture, although the level of protective immunity induced by different vaccines varies greatly [19,33]. DNA vaccines in fish are administered mainly by intramuscular injection and consist of a recombinant plasmid encoding a viral antigenic protein [21]. In the present study, a DNA vaccine that includes the LCDV-Sa *mcp* gene was constructed. MCP has been identified as a highly immunogenic protein in different species of iridoviruses [34,35,36]. Furthermore, the *mcp* gene has been used in other DNA vaccines against several species of the *Iridoviridae* family, showing its protective capacity against LCDV-C [23,37], the red sea bream iridovirus (RSIV) [38] or the infectious spleen and kidney necrosis virus (ISKNV) [39].

Several studies have analyzed the persistence of the plasmid DNA and its expression capacity in vaccinated animals, showing that they are more stable in fish than in mammals [40,41,42]. In gilthead seabream, pcDNA-MCP persists for at least 20 days, presenting a systemic distribution. Moreover, *mcp* transcripts were consistently detected in HK, suggesting its role as target organ for vaccine expression, as demonstrated in other studies [41,43]. The transcription of the viral gene encoded in the vaccine decreased over time in the muscle at the site of the injection, which may be due to the quick spread of plasmid DNA in small fish as other studies have pointed out [44,45,46].

Viral load and gene expression after LCDV-Sa challenge in vaccinated and mock-vaccinated, (i.e., injected with the empty plasmid) gilthead seabream was analyzed by qPCR in order to evaluate the protective capacity of the vaccine. Viral transcripts were detected only in 16.7% of the vaccinated fish at 10 dpi, whereas all control fish were infected and 83.3% of them supported a productive infection. Even though a reduced number of animals were used in the vaccination trial, the results obtained suggest that the vaccine produces a reduction in the viral load during the course of infection, or at least limits viral multiplication. These results are similar to those previously reported for other viral DNA vaccines in fish [38,47].

It is well known that DNA vaccination can induce or enhance the expression of various immune-related genes in fish [21,22]. Overall, the initial response after vaccination is triggered by antigen-presenting cells (APC) [48], allowing the presentation of the antigenic peptide encoded in the vaccine by either MHC class I or MHC class II molecules. The T-cells receptor (TCR) may recognize those peptides, stimulating cytotoxic T cells (CD8^+^) and helper T-cells (CD4^+^) [42].

In this study, the transcriptional profile of 23 immune-related genes in two organs, the HK and intestine, was analyzed to study the immune response of gilthead seabream after DNA vaccination. The genes included were those encoding two TLR (*tlr5* and *tlr9*), members of type I INF pathway (*ifn, irf1, irf3, irf9, pkr, mx1, mx2, mx3* and *isg15*), cytokines related to the inflammation process (*tnfα, il1β, il6* and *il10*), the pro-inflammatory caspase 1 (*casp1*), chemokines (*ck3* and *ck10)*, the complement component C3 (*c3*), and receptor markers characteristic of non-specific cytotoxic cells (*nccrp1*), antigen-presenting cells (*mhcIIα*) and T and B lymphocytes (*tcrβ* and *ighm*, respectively), that were previously used to evaluate the immune gene expression response of gilthead seabream experimentally infected with LCDV-Sa [26].

In mammals, the immune system activation driven by exogenous DNA relies on DNA recognition by TLR, more specifically TLR9. Mammalian TLR9 recognizes plasmid DNA that contains short sequences of unmethylated CpG dinucleotides [49]; those motifs are found in the vaccine DNA and exert an immune-stimulant effect [50]. In vaccinated gilthead seabream, the expression of the *tlr9* gene was up-regulated, and a temporal coincidence was observed with the expression level of the *mcp* gene contained in the vaccine. Moreover, the empty plasmid also produces an up-regulation of *tlr9* in HK, which supports that fish TLR9 can bind to plasmid DNA.

Induction of type I IFN system is a key component in the antiviral innate immunity. IRFs 1 and 3 are positive regulators of type I IFN gene transcription, while IRF9 participates in the IFN signaling pathway [51]. In vaccinated fish an early down-regulation of *irf1* and *irf3* expression was observed, whereas the expression of *irf9* was significantly activated. This activation was also observed in animals injected with empty plasmid, but activation was significantly lower. The *ifn* gene was activated in the HK and intestine at 1 and 8 dpv, respectively, although in the empty plasmid group its expression was also up-regulated at the same level. Regarding the expression of IFN-stimulated genes, a similar regulation was observed in vaccinated and mock-vaccinated fish. Thus, there was a down-regulation of *mx1, mx2* and *pkr* in the HK, and of *isg15* in the intestine, while the *mx3* gene was activated in the HK at 8 dpv and in the intestine at 1 dpv. Interestingly, the expression of *mx3* in the intestine was significantly higher in mock-vaccinated fish. It has to be noted that gilthead seabream Mx1 and Mx2 isoforms, but not Mx3, show in vitro activity against LCDV-Sa [52]. Therefore, unlikely to what occurred in fish infected by LCDV [26,53], the vaccine does not induce the differential overexpression of genes related to the innate antiviral response.

In addition, the DNA vaccine triggers an overexpression of pro-inflammatory genes. There was an early activation in the HK and/or intestine of *il1β, il6* and *casp1,* as well as chemokines *ck3* and *ck10*. However, in the fish injected with the empty plasmid these genes appear un-affected or down-regulated. In the case of the *il10* gene, there was a significant deactivation in the HK at 3 dpv with respect to the empty plasmid group. The results obtained in a previous study in gilthead seabream experimentally infected with LCDV-Sa showed a lack of induction of pro-inflammatory cytokines together with an early overexpression of *il10*, which may be related to the establishment of a persistent infection [26]. The DNA vaccine may have the opposite effect, inactivating the expression of *il10* and activating genes related to the inflammatory process. Thus, the vaccine appears to induce a systemic inflammatory response in gilthead seabream.

The complement component C3 plays a key role in the activation pathways of the complement system, helping to coordinate the downstream immune response, triggering inflammation and immune clearance [54,55]. In the present study, a strong induction of *c3* transcription was observed in the HK and intestine, which corresponds to the highest expression values detected. These results have also been reported in Japanese flounder and gilthead seabream in response to LCDV infection, indicating that it could be a common host defense strategy triggered by LCDV infection [26,56]. This would imply that the DNA vaccine is able to trigger the activation of the complement system, which could build a defensive strategy against further viral infections since the complement system is involved in both innate and adaptive immune responses, being able to induce an inflammatory response.

Finally, with all the genes analyzed, coding receptor markers of immune cells were up-regulated in the intestine of vaccinated fish, although none of them were differentially expressed in the HK. At 3 dpv an up-regulation of *nccrp1* and *mhcIIα* was observed, whereas the markers of T and B lymphocytes (*tcrβ* and *ighm*, respectively) appeared up-regulated at the end of the experimental period. This may suggest that the vaccine results in mucosal immunity, an important factor in preventing infection [57]. In Japanese flounder vaccinated against LCDV-C the expression of *tcrβ* and *mhcIIα* genes was up-regulated both in the HK and intestine [58]. Our results could indicate that the vaccine triggers an immune response in gilthead seabream in which antigen-presenting cells, and T and B lymphocytes are induced in the intestinal mucosa.

Viral recognition and neutralization by specific antibodies are essential for the antiviral immune response, participating in the eventual clearance of a virus from the body [46]. The detection of specific antibodies has been observed in fish recovered from LCD [unpublished results]. In vaccinated gilthead seabream, an efficient humoral immune response was induced, as demonstrated by the high titer of specific anti-MCP antibodies determined by ELISA in fish at 3 months post-vaccination. This humoral response has also been observed in vaccinated Japanese flounder [25,58]. However, to our knowledge, the presence of neutralizing antibodies were proved for the first time.

In summary, the pcDNA-MCP vaccine administrated intramuscularly to gilthead seabream triggers an immune response characterized by the overexpression of genes related to the inflammatory process and others related with an activation of the adaptive humoral immune response, as demonstrated by the production of neutralizing antibodies. This response may explain the protection against LCDV-Sa infection observed in vaccinated fish. Although further studies must be performed to establish the protective capacity of the vaccine in field challenges, our results support the potential of this DNA vaccine in controlling lymphocystis disease in gilthead seabream aquaculture.

## Figures and Tables

**Table 1:** Primers designed for DNA vaccine construction and detection.

Assay	Primers	Sequence (5′–3′)	Amplicon Size
*mcp* cloning	MCP-F	AAGCTAGCTATGACTTCTGTAGCGG	1398 bp
	MCP-R	TATCTAGATCTACAACACAGGGAAACCC	
PCR	pcDNA-MCP-F	CGATTTGGTGGCTCAAAAAT	767 bp
	pcDNA-MCP-R	CTGTTTCTACGGGGATGGAA	
nPCR	pcDNA-MCP-nF	ATATGACGCAACCCGTTGAT	224 bp
	pcDNA-MCP-nR	TTCTAAATCTCCCGCCGTTA	

Primers were designed using the sequence of the LCDVSa062R ORF (GenBank accession number KX643370.1) and Primer3web version 4.1.0 (http://bioinfo.ut.ee/primer3/) (accessed on 19 July 2017). Start and stop codons in MCP primers were underlined.

**Figure A1:**
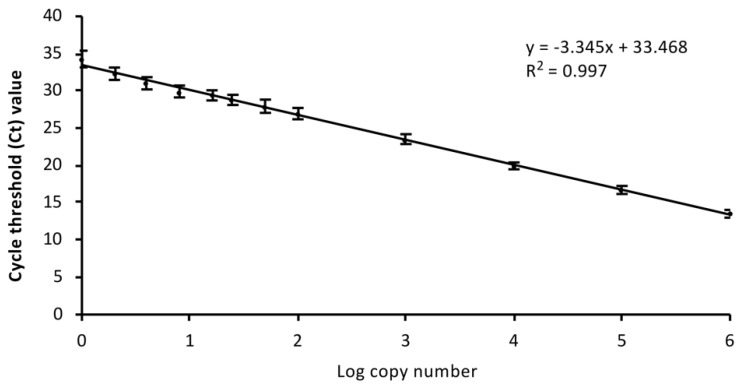
Standard curve obtained using dilutions of the plasmid DNA ranging from 10^6^ to 2 copies per reaction. Linear regression was performed on mean data from three separate runs.

## Data Availability

The data presented in this study are available on request from the corresponding author.

## Supplementary Materials

The following are available online at https://www.mdpi.com/article/10.3390/ani11061613/s1, Table S1: Relative expression and *p*-value of immune-related genes in head kidney samples from vaccinated and mock-vaccinated fish at different post-vaccination times, Table S2: Relative expression and *p*-value of immune-related genes in intestine samples from vaccinated and mock-vaccinated fish at different post-vaccination times.

## Appendix A

The detection and quantification of viral DNA in vaccinated fish challenged with LCDV-Sa were carried out by a qPCR assay targeting a viral structural gene alternative to the *mcp* gene contained in the vaccine. LCDVSa074R ORF (GenBank accession number KX643370.1), which encoded a putative myristoylated membrane protein (MMP) consisting of 307 amino acids [11], was the target gene selected.

A 416-bp fragment of the *mmp* gene was amplified by PCR using the primer set specified in Table A1, cloned into the pCR2.1-TOPO vector (Invitrogen) and subsequently used to transform *E. coli* One Shot TOP10 cells. Recombinant plasmid DNA was purified from *E. coli* cells with the High Pure Plasmid Isolation Kit (Roche Diagnostics), and insert size was verified by PCR and sequencing, using the M13 primers provided in the TOPO TA Cloning Kit (Invitrogen). Purified recombinant plasmid was stored at −20 °C until used.

**Table A1:** Primers designed for viral DNA quantification by real-time PCR.

Assay	Primers	Sequence (5′–3′)	Amplicon Size
*mmp* cloning	MMP-PCR-F	ATTTAGCACGCGCTTCAGAT	416 bp
	MMP-PCR-R	TTTTATGGGCGGTTTTTCAG	
qPCR	MMP-qPCR-F	TTGCCCCACTTCCTATTGTC	122 bp
	MMP-qPCR-R	CCGGTTTTTCAGACTTGGAA	

Primers were designed using the sequence of the LCDVSa074R ORF (GenBank accession number KX643370.1) and Primer3web version 4.1.0 (http://bioinfo.ut.ee/primer3/) (accessed on 19 July 2017).

Primers for qPCR (Table A1) generate a 122-bp amplicon within the 416-bp cloned fragment present in the recombinant plasmid. Real-time PCR reactions were carried out in a final volume of 20 µL containing 12.5 µL of FastStart Essential DNA Green Master (Roche Diagnostics), 2 µL of each primer (10 pmol/µL), and 200 ng of DNA. PCR amplifications were performed in a LightCycler^®^ 96 Instrument (Roche Diagnostics). The thermal profile was: 95 °C for 10 min, and 40 cycles at 95 °C for 10 s, 60 °C for 10 s and 72 °C for 10s. Finally, dissociation curve analysis was carried out automatically to detect non-specific amplification products.

To quantify the amount of viral DNA in different samples, a standard curve was generated using the recombinant plasmid described above. The concentration of the purified plasmid was determined by spectrophotometry (NanoDrop 1000), and the plasmid stock was diluted to serve as template for qPCR (dilutions ranging from 10^6^ copies to 1 copy). In each 96-well, plasmid dilutions for the standard curve were ran along with the samples and controls (Milli-Q water and DNA from one LCDV-negative gilthead seabream sample), using three technical replicates. The number of copies of viral DNA in each well was calculated from its cycle threshold (Ct) value by interpolation in the standard curve (Ct versus log copy number). The amplification efficiency (E) was calculated from standard curves using the formula E = (10^−1/S^ − 1) × 100 (S being the slope of the linear fit). Viral loads in samples were calculated as the mean of the three replicates.

To evaluate the precision of the qPCR, the intra- and inter-assay variability was determined using the recombinant plasmid. To assess intra-assay variation, three plasmid DNA dilution series were prepared and tested simultaneously in the same plate, whereas three separate PCR runs were carried out to assess inter-assay variation, using the same dilutions of plasmid DNA. The mean, standard deviation (SD) and coefficient of variation (CV) were calculated independently for each DNA dilution.

## Appendix B

Specificity of the qPCR assay was determined by analysis of the dissociation curves obtained in each experiment. Standard and positive samples gave a single PCR product with a melting temperature of 78.0 ± 0.5 °C and the expected size, as observed in agarose gel electrophoresis.

The linear dynamic range, efficiency and precision of the qPCR assay were evaluated using a recombinant plasmid containing a 416-bp fragment of the *mmp* gene. A linear relationship between the amount of plasmid DNA and Ct values was demonstrated, using standard curves generated from three independent assays. The linear range of the assay was from 10^6^ to 2 copies per reaction (Figure A1). The regression analysis yielded a correlation coefficient (*r*) ≥ 0.998 and E value of 99.03 ± 3.53%.

The mean intra-assay variation was 2.63 ± 0.65% when analyzing three replicates of plasmid dilutions, whereas the mean inter-assay variation among three independent experiments was 2.57 ± 0.60% (Table A2). These CV values were considered acceptable to validate the repeatability and reproducibility of the assay.

**Table A2:** Intra- and inter-assay variability of the real-time PCR.

	Intra-Assay Variation ^1^		Inter-Assay Variation ^2^	
Copies/Reaction	Cycle Threshold (C_t_) ^3^	CV (%)	Cycle Threshold (C_t_) ^3^	CV (%)
1 × 10^5^	16.78 ± 0.54	3.21	16.39 ± 0.42	2.56
1 × 10^4^	20.03 ± 0.55	2.74	19.78 ± 0.32	1.61
1 × 10^3^	23.63 ± 0.80	3.38	23.26 ± 0.44	1.80
1 × 10^2^	27.38 ± 0.86	3.14	26.55 ± 0.26	0.97
5 × 10^1^	28.43 ± 0.68	2.39	27.47 ± 0.85	3.09
2.5 × 10^1^	28.98 ± 0.83	2.86	28.47 ± 0.46	1.61
1.6 × 10^1^	29.13 ± 0.90	3.08	29.17 ± 0.80	2.74
8 × 10^0^	30.40 ± 0.54	1.77	29.48 ± 0.65	2.20
4 × 10^0^	31.63 ± 0.76	2.40	30.03 ± 0.59	1.96
2 × 10^0^	32.94 ± 0.45	1.36	31.96 ± 0.65	2.03
Overall CV ^3^ (%)		2.63 ± 0.65		2.57 ± 0.60

^1^ Intra-assay variation was calculated on three replicates of recombinant plasmid dilutions analyzed in the same PCR run. ^2^ Inter-assay variation was calculated on values obtained in three separate PCR runs. ^3^ Mean ± standard deviation (SD). CV: coefficient of variation.
