# Supplementary material for: Evaluation of Gilthead Seabream (Sparus aurata) Immune Response after LCDV-Sa DNA Vaccination
Source: Animals (Basel). 2021 May 29;11(6):1613. doi: 10.3390/ani11061613 (PMC8228267; doi:10.3390/ani11061613)
Supplement: Supplementary file 1 [file animals-11-01613-s001.zip › animals-1225635-supplementary/Tables S/Table S1.pdf]

Table S1. Relative expression and *p*-value of immune-related genes in head kidney samples from vaccinated and mock-vaccinated fish at different post-vaccination times.

	1 dpv			3 dpv			8 dpv		
Gene	pcDNA-MCP	pcDNA	*p*-value	pcDNA-MCP	pcDNA	*p*-value	pcDNA-MCP	pcDNA	*p*-value
tlr5	0.49 ±0.10*	0.84 ±0.30	0.3109	0.58 ±0.36	4.12 ±0.98*	**0.0086**	1.37 ±0.91	0.52 ±0.09*	0.4046
tlr9	1.30 ±0.14	1.44 ±0.62	0.5076	0.91 ±0.46	0.58 ±0.16	0.7480	20.80 ±14.14*	5.17 ±1.91*	0.5403
ifn	2.01 ±0.55*	1.90 ±0.39	0.8657	1.57 ±0.63	1.17 ±0.13	0.8589	3.00 ±1.39	1.90 ±0.46	0.9205
irf1	2.06 ±0.49	1.60 ±0.31	0.4631	2.48 ±1.07	0.69 ±0.07	0.0538	1.91 ±0.94	0.36 ±0.07	0.2898
irf3	0.93 ±0.09	2.57 ±0.89	**0.0147**	0.54 ±0.05*	4.51 ±2.12*	**0.0327**	1.16 ±0.45	0.64 ±0.06	0.5349
irf9	14.17 ±3.34*	2.14 ±1.01	**0.0012**	1.58 ±0.23*	1.38 ±0.64	0.3356	0.17 ±0.07*	0..34 ±0.05*	0.0996
pkr	1.72 ±0.36	1.53 ±0.37	0.7230	1.11 ±0.37	0.97 ±0.41	0.9743	0.37 ±0.18*	1.38 ±0.38	0.9112
mx1	0.53 ±0.09*	0.97 ±0.12	**0.0459**	0.45 ±0.06*	0.54 ±0.26	0.3669	0.62 ±0.25	0.73 ±0.04	0.2873
mx2	1.23 ±0.37	1.26 ±0.19	0.7379	0.76 ±0.07	0.49 ±0.07	**0.0371**	0.08 ±0.06*	0.59 ±0.02	**0.0015**
mx3	3.02 ±0.61	1.70 ±1.86*	0.7617	0.69 ±0.06*	0.19 ±0.02	**0.0001**	17.01 ±8.45*	0.16 ±0.01*	**0.0001**
isg15	1.26 ±0.20	1.68 ±0.54	0.8073	0.94 ±0.25	1.92 ±0.90	0.7210	1.69 ±0.82	0.52 ±0.28	0.2621
tnfα	0.34 ±0.08*	0.73 ±0.12*	**0.0372**	1.25 ±0.39	0.97 ±0.11	0.8928	10.99 ±8.02	0.51 ±0.13*	0.0795
casp1	1.43 ±0.29	1.20 ±0.15	0.6392	0.69 ±0.49	0.46 ±0.10*	0.3227	1.38 ±0.84	0.76 ±0.13	0.6870
il1β	2.76 ±1.30	0.05 ±0.01*	**0.0001**	12.14 ±2.50*	0.38 ±0.13*	**0.0001**	1.31 ±0.52	0.52 ±0.22	0.2168
il6	1.02 ±0.25	0.02 ±0.00*	**0.0001**	28.39 ±11.65*	0.32 ±0.04*	**0.0001**	3.69 ±1.84	0.82 ±0.30	0.1329
il10	0.71 ±0.17	1.61 ±0.19	**0.0165**	0.26 ±0.02*	0.74 ±0.09	**0.0006**	2.04 ±0.94	1.16 ±0.16	0.9079
ck3	4.19 ±1.67*	1.02 ±0.30	**0.0188**	2.50 ±0.73	0.34 ±0.08*	**0.0016**	3.54 ±2.10	0.56 ±0.11	**0.0290**
ck10	4.04 ±1.69*	0.81 ±0.32	**0.0303**	1.91 ±0.97	0.32 ±0.08*	**0.0185**	0.33 ±0.21*	0.09 ±0.01*	0.6588
c3	2.08 ±1.17	0.49 ±0.21	0.2334	309.41 ±290.65*	0.30 ±0.14*	**0.0051**	4.41 ±2.29	0.72 ±0.27	0.6448
nccrp-1	0.99 ±0.23	0.75 ±0.26	0.3314	0.86 ±0.40	0.40 ±0.08*	0.8254	3.56 ±1.84	0.75 ±0.22	0.1341
tcrβ	1.48 ±0.42	0.82 ±0.25	0.2108	1.29 ±0.35	0.16 ±0.09*	**0.0030**	2.17 ±0.99	0.07 ±0.008*	**0.0006**
ighm	3.07 ±1.44	1.95 ±0.51	0.4754	0.80 ±0.33	1.17 ±0.13	0.1803	1.31 ±0.63	1.92 ±0.49	0.3121
mhcIIα	1.69 ±0.39	2.66 ±1.00	0.3524	1.15 ±0.60	0.90 ±0.09	0.6042	0.93 ±0.45	0.75 ±0.08	0.5573

Relative expression levels ($2^{-\Delta\Delta Ct}$) data are expressed as mean ± SEM (n = 5). Asterisks denote differentially expressed genes (DEGs) compared to the control group injected with PBS. Significant differences between vaccinated (pcDNA-MCP) and mock-vaccinated (pcDNA) groups ($p < 0.05$) are indicated in bold.
