# Supplementary material for: Evaluation of Gilthead Seabream (Sparus aurata) Immune Response after LCDV-Sa DNA Vaccination
Source: Animals (Basel). 2021 May 29;11(6):1613. doi: 10.3390/ani11061613 (PMC8228267; doi:10.3390/ani11061613)
Supplement: Supplementary file 1 [file animals-11-01613-s001.zip › animals-1225635-supplementary/Tables S/Table S2.pdf]

Table S2. Relative expression and *p*-value of immune-related genes in intestine samples from vaccinated and mock-vaccinated fish at different post-vaccination times.

	1 dpv			3 dpv			8 dpv		
Gene	pcDNA-MCP	pcDNA	*p*-value	pcDNA-MCP	pcDNA	*p*-value	pcDNA-MCP	pcDNA	*p*-value
tlr5	1.77±0.57	0.77±0.08	**0.0391**	1.31±0.27	1.11±0.60	0.2793	2.352±0.15*	0.81±0.11	**0.0001**
tlr9	2.57±0.85	1.55±0.29	0.4258	1.67±0.20*	0.50±0.04*	**0.0001**	1.71±0.39	1.38±0.29	0.5212
ifn	1.91±0.54	1.30±0.34	0.3463	1.39±0.30	1.52±0.40	0.9296	5.44±1.04*	6.58±1.69*	0.7676
irf1	0.03±0.02*	1.73±0.48	**0.0001**	0.08±0.05*	0.48±0.25	0.0536	7.24±5.49	2.06±0.50	0.5013
irf3	0.58±0.14*	0.91±0.21	0.5700	0.66±0.13	1.54±0.97	0.5341	0.77±0.14	1.03±0.08	0.1189
irf9	0.91±0.22	1.83±0.51	0.1239	4.39±1.15*	2.43±1.15	0.2603	1.48±0.30	1.31±0.31	0.6561
pkr	1.46±0.37	0.88±0.18	0.2099	1.44±0.30	0.24±0.12*	**0.0083**	1.83±0.22*	1.03±0.20	0.0849
mx1	1.72±0.45	2.52±0.33	0.1821	0.80±0.30	0.26±0.03*	0.1186	1.24±0.46	2.42±0.45	0.0766
mx2	1.90±0.60	2.12±0.35	0.4933	0.84±0.34	0.20±0.05*	0.0726	1.96±0.77	2.56±0.67	0.4276
mx3	5.34±0.99*	24.59±5.59*	**0.0012**	0.50±0.25	3.65±0.12	0.2612	2.03±0.26	0.30±0.69	**0.0370**
isg15	0.65±0.20*	1.11±0.48	0.8819	0.72±0.35	0.48±0.23	0.4965	1.14±0.40	1.08±0.40	0.6206
tnfα	0.59±0.19*	1.52±0.44	0.0947	1.72±0.33	0.93±0.22	0.0812	1.97±0.34*	1.33±0.38	0.1785
casp1	1.35±0.50	1.43±0.27	0.5620	1.76±0.23*	0.49±0.12*	**0.0027**	1.98±0.24*	1.37±0.12	**0.0296**
il1β	11.34±7.26*	1.11±0.22	**0.0286**	46.41±22.71*	4.92±0.65	**0.0093**	0.87±0.34	0.56±0.19	0.9943
il6	4.90±2.63*	0.83±0.23	**0.0335**	9.10±2.28*	3.11±1.39	**0.0287**	1.97±0.32*	1.66±0.46	0.4452
il10	1.16±0.44	0.747±0.19	0.5786	1.66±0.46	0.86±0.13	0.2487	1.76±0.26*	2.71±0.98	0.7259
ck3	1.80±1.12	1.03±0.24	0.9939	4.07±1.43	1.12±0.14	0.0565	1.35±0.24	0.40±0.13*	**0.0082**
ck10	2.39±1.65	1.20±0.36	0.7911	6.09±1.43*	0.51±0.21	**0.0014**	4.58±2.30*	0.91±0.19	0.0699
c3	9.88±7.33	0.88±0.10	0.1128	8.27±1.30*	3.76±0.97*	**0.0408**	553.65±180.56*	36.10±5.16	**0.0021**
nccrp-1	4.90±3.15	0.83±0.24	0.0774	32.22±9.44*	1.08±0.28	**0.0003**	1.05±0.11	0.59±0.07*	**0.0096**
tcrβ	2.11±0.75	1.44±0.28	0.5819	0.86±0.22	0.55±0.11	0.4898	2.43±0.18*	1.27±0.23	**0.0139**
ighm	1.37±0.63	0.59±0.27	0.2607	1.11±0.16	0.16±0.07	**0.0018**	3.18±0.20*	1.04±0.74	**0.0023**
mhcIIα	2.62±1.06	1.50±0.34	0.4709	26.85±11.28*	3.47±0.42	**0.0021**	2.86±0.70*	2.14±0.47*	0.6534

Relative expression levels ($2^{-\Delta\Delta Ct}$) data are expressed as mean ± SEM (n = 5). Asterisks denote differentially expressed genes (DEGs) compared to the control group injected with PBS. Significant differences between vaccinated (pcDNA-MCP) and mock-vaccinated (pcDNA) groups ($p < 0.05$) are indicated in bold.
